# THE IMPACT OF “TELE-SAVVY” ON THE CAREGIVING EXPERIENCE OF DEMENTIA FAMILY CAREGIVERS: QUALITATIVE FINDINGS

**DOI:** 10.1093/geroni/igz038.3196

**Published:** 2019-11-08

**Authors:** Fayron Epps, Elizabeth Maloch, Patricia Griffiths, Ken Hepburn

**Affiliations:** 1 Emory University, Atlanta, Georgia, United States; 2 Emory University School of Medicine, Atlanta, Georgia, United States

## Abstract

Tele-Savvy is a 3-arm randomized controlled trial (RCT) of a psychoeducation intervention that equips family caregivers of people living with dementia with the knowledge and skills they need to provide care to their person, while also caring for themselves. This RCT is currently underway, with cohorts rotating through over a period of 12 months. The purpose of this presentation is to describe the effectiveness of Tele-Savvy (active) versus Healthy Living Intervention (attention control) or usual care (wait-list) on the caregiving experience among dementia family caregivers. We conducted semi-structured interviews with 16 caregivers at the 6 month time point and after their initial participation in either the active, attention control, or usual care groups. Interviews elicited caregivers’ perceptions regarding the program’s influence on their caregiving experience. Caregivers who participated in Tele-Savvy reported positive experiences such as recognizing that their experience has become more pleasurable and happier. They also shared that the program expanded their knowledge and allowed them to become patient while implementing caregiving strategies learned. Majority of caregivers who participated in the Healthy Living program indicated that nothing changed related to their caregiving experience. Half of the caregivers in the usual care group, expressed frustration and unhappiness, while the others expressed no change in their caregiving experience. Results suggest that psychoeducation caregiver interventions are meaningful and can enact positive changes in their outlook on caregiving. Thus, results of this study may guide future policies and further development of caregiver programs and interventions.

